# Mesenchymal Stem Cells in Fungal Infections: Immunomodulation, Direct Antifungal Activity, and the Promise of the Secretome

**DOI:** 10.3390/biomedicines14050994

**Published:** 2026-04-27

**Authors:** Maya Nehemia, Hilit Cohen, Orly Gruzman, Tal Meushar Vega Amador, Shimon B. Levy, Sorina Grisaru-Granovsky, Ofra Ben Menachem-Zidon

**Affiliations:** 1 Hanan Drori Regenerative Medicine Research Laboratory, Department of Obstetrics & Gynecology, The Eisenberg R&D Authority, Shaare Zedek Medical Center, Jerusalem 9103102, Israel; 2 Department of Obstetrics & Gynecology, Shaare Zedek Medical Center, Jerusalem 9103102, Israel; 3 The Institute for Medical Research Israel-Canada (IMRIC), The Hebrew University of Jerusalem, Jerusalem 9112102, Israel

**Keywords:** mesenchymal stem cells, secretome, macrophages, invasive fungal infections, immunomodulation, direct antifungal activity

## Abstract

Mesenchymal stem/stromal cells (MSCs) are widely recognized as potent modulators of inflammation and immune function in bacterial and viral infections. However, their roles in fungal disease remain comparatively under-defined despite the growing clinical burden of invasive and opportunistic mycoses. This Feature Review synthesizes emerging evidence that MSCs influence antifungal outcomes through two complementary axes: (i) host-directed effects, including modulation of immune responses, particularly macrophage responses, and tissue/barrier conditioning; and (ii) fungus-directed effects (direct antifungal activity mediated by contact-dependent mechanisms and secreted antimicrobial factors). We will summarize how MSCs reshape cytokine and chemokine networks and tune innate immune effector functions, with emphasis on macrophage polarization, pattern-recognition receptor signaling, and downstream phagocytic and fungicidal pathways. In parallel, we will review data suggesting that MSCs can interact more directly with fungal pathogens through sensing, physical engagement, and secretion of antimicrobial mediators while highlighting mechanistic uncertainties and model-dependent limitations. A dedicated section will address MSC-derived secretome products (conditioned media, extracellular vesicles) as a cell-free strategy to enhance antifungal immunity. We will critically evaluate conflicting findings across studies, highlighting that outcomes depend on pathogen and host context. Clarifying these context dependencies is essential to rationally develop MSC or secretome-based interventions that are safe, reproducible, and tailored to specific fungal pathogens and patient populations.

## 1. Introduction

Invasive fungal infections (IFIs), including invasive mold infections (IMIs), remain a leading cause of morbidity and mortality in immunocompromised populations [1]. Despite advances in antifungal therapy, outcomes often remain suboptimal, due to delayed diagnosis, drug toxicity, emerging resistance [2,3,4,5] and dysregulated host inflammatory response. Disease severity reflects both fungal burden and maladaptive immune activation, consistent with the damage–response framework in which both insufficient and excessive inflammation contribute to pathology [6,7,8,9,10,11,12,13]. Within this framework, early innate immunity, particularly macrophage function, is thought to play a central role in shaping disease trajectory. As first responders, macrophages help to contain fungal growth and orchestrate downstream effector recruitment. However, opportunistic pathogens like *Candida albicans* can exploit macrophages as intracellular niches, promoting persistence and dissemination while including damaging inflammatory programs by enabling intracellular survival and impairing intracellular killing mechanism [10,14,15]. Depending on the immunological context and activation state, macrophage responses may either promote clearance or permit persistence. This duality highlights a central therapeutic challenge: how to recalibrate macrophage responses to preserve antifungal defense while limiting collateral tissue injury.

This has driven interest in host-directed therapies, including mesenchymal stem cells (MSCs), which exhibit context-dependent immunomodulatory and tissue-reparative properties, largely mediated through paracrine mechanisms. Increasing focus toward the MSC secretome, comprising soluble factors and extracellular vesicles, suggests a potentially more controllable therapeutic approach, although standardization remains challenging. Unlike whole-cell therapies, the secretome is associated with reduced risks of unwanted differentiation, ectopic tissue formation, and tumorigenicity. It may represent a potentially safer option for vulnerable patient populations [16,17,18,19,20,21].

Here, we evaluate MSCs through a damage–response lens, focusing on how MSC–macrophage crosstalk may recalibrate inflammatory set-points such as the TNF/IL-10 balance and NF-κB-associated signaling pathways, enhance phagocytic and pro-resolving functions, and influence the balance between fungal control and tissue injury. We synthesize evidence for direct MSC–fungus interactions and reconcile divergent outcomes across experimental systems to highlight the secretome as a potential viable therapeutic strategy. Finally, we consider the high-stakes clinical intersection of graft-versus-host disease (GvHD)—a setting tightly linked to invasive fungal infections due to profound immune dysfunction and immunosuppressive therapy—where MSCs are already administered as a treatment. Therefore, clarifying how MSC exposure shapes concurrent fungal infection risk is critical for strengthening causal inference around both safety and efficacy [1].

At the same time, the literature directly examining MSC effects in fungal infection remains limited. Interpretation is further complicated by the absence of a single ideal model for studying these interactions, as each system captures only part of the relevant biology. In vitro models provide controlled mechanistic insight, whereas in vivo studies and clinical observations reflect additional layers of host complexity and translational uncertainty. In this review, we therefore distinguish as clearly as possible between in vitro, in vivo, and clinical evidence, including data derived from GvHD patients, in order to separate direct experimental findings from broader clinical associations [22].

## 2. Immunological Landscape of Fungal Infections

### 2.1. Innate Immune Recognition of Fungi

Innate immune recognition of fungi is mediated by pattern recognition receptors (PRRs) that detect conserved cell wall components such as β glucans, mannans, and chitin [22,23,24,25,26]. Macrophages play a central role in this process, coupling phagocytic uptake with the orchestration of inflammatory and antimicrobial programs [22,27,28,29]. Recognition is coordinated through multiple PRR families, including C-type lectin receptors (e.g., Dectin 1), Toll-like-receptors (e.g., TLR2, TLR4), and complement receptors, enabling context-dependent detection of diverse fungal pathogens [8,14].

Antifungal immunity involves combinatorial PRR engagement, whereby different fungal species and morphologies activate multiple receptors simultaneously, generating integrated signaling outputs that shape transcriptional and inflammatory responses [24]. These signals converge on pathways such as NF-κB, MAPK cascades, and inflammasome pathways, regulating cytokine production and effector cell recruitment [30,31].

PRR engagement does not produce a uniform outcome. Host responses are influenced by fungal morphology, inoculum size, and immune context, leading to either effective clearance or sustained inflammation that contributes to tissue damage [8,14]. Morphological transitions—such as yeast to hyphae in *Candida albicans* or conidia to hyphae in *Aspergillus fumigatus*—alter ligand exposure and reshape immune activation [30,32]. Thus, consistent with the damage–response framework, fungal recognition can promote either protection or host-mediated pathology.

### 2.2. Macrophages as Central Regulators of Fungal Immunity

Macrophages are central effectors of antifungal immunity, integrating pathogen recognition, phagocytosis, and orchestration of early inflammatory responses [8,14,28,33]. Through PRR engagement, including C-type lectin receptors and Toll-like receptors, they initiate antimicrobial and inflammatory programs that shape early infection trajectory [34,35].

This sentinel role is particularly evident in pulmonary infection with *Aspergillus fumigatus*. Following inhalation of conidia, alveolar macrophages rapidly internalize spores and initiate transcriptional programs that promote neutrophil recruitment, establishing the first layer of antifungal defense [33,36]. The efficiency of this early containment influences whether infection remains localized or progresses toward invasive disease.

Phagocytosis acts as a regulatory checkpoint in antifungal immunity: successful engulfment and intracellular killing may support containment and resolution, whereas failed or frustrated phagocytosis can sustain inflammatory signaling, inflammasome activation, and tissue injury [8,31]. Thus, the quality of the phagocytic encounter determines whether macrophages promote resolution or amplify inflammation.

Macrophage responses are further shaped by fungal species and morphology. In *Candida albicans*, cell wall glycosylation influences recognition efficiency and macrophage recruitment [37], whereas in *Aspergillus fumigatus*, dormant conidia are typically internalized with limited signaling, while germinating hyphae induce stronger inflammatory responses, including IL-1β- and inflammasome-associated activation [30,32].

Hyphae-induced inflammation has been shown to involve NLRP3 inflammasome activation, requiring Syk signaling, potassium efflux, and ROS generation, while MyD88-dependent pathways regulate IL-1β transcription [29,30]. Importantly, heightened inflammatory activation does not necessarily correlate with improved fungal clearance. In both *Candida* and *Aspergillus* models, macrophage activation can become uncoupled from fungicidal capacity, resulting in tissue damaging inflammation without effective pathogen elimination [30,31,32,38]. These dynamics place macrophages at the center of the damage–response continuum [10,39,40,41].

### 2.3. Macrophages as Intracellular Niches for Fungi

Macrophage engagement does not uniformly culminate in fungal elimination. Across pathogens, macrophages can function as permissive intracellular niches that may support survival, immune evasion, and dissemination. Rather than acting solely as effector cells, macrophages may become sites of incomplete killing, altered inflammatory programming, and pathogen adaptation [8,10,22,42,43,44,45]. Intracellular residence shields fungi from extracellular immunity and antifungal drugs while enabling escape, replication, and spread.

*Candida albicans* exemplifies this duality. Following phagocytosis, *Candida* can persist within macrophages due to impaired intracellular killing and continued morphogenesis that facilitates escape [37,46]. Mechanistically, experimental studies have shown that *Candida* disrupts autophagic processes, including LC3 turnover, limiting effective degradation [46].

Escape is often associated with macrophage damage mediated by inflammasome activation and pore-forming pathways. For example, gasdermin D deficiency reduces fungal escape, whereas NLRP3 inhibition increases macrophage death without preventing escape, illustrating how intracellular persistence can amplify inflammation and tissue injury [47].

Importantly, excessive attenuation of inflammation is not uniformly protective. During *Candida* infection, macrophages produce IL 1 receptor antagonist (IL 1Ra), which suppresses neutrophil recruitment and compromises fungal clearance [48]. Thus, both impaired killing and dysregulated inflammatory control can sustain persistence, with outcomes varying depending on the balance between intracellular killing efficiency and inflammatory regulation.

A parallel strategy is evident in *Cryptococcus neoformans*, which survives and replicates within macrophages by resisting intracellular killing and modulating phagolysosomal maturation [43,49,50]. Its hallmark non-lytic exocytosis enables fungal egress without host cell lysis, preserving both pathogen viability and macrophage integrity and allowing repeated cycles of intracellular residence and dissemination [42].

*Histoplasma capsulatum* similarly supports intracellular persistence by altering macrophage transcriptional and post-transcriptional networks (including MAPK, TGF-β, Wnt, and p53 pathways) and cytoskeletal pathways through coordinated changes in macrophage miRNA profiles [51,52,53], impairing phagolysosomal maturation and antimicrobial functions, and reinforcing the establishment of a permissive intracellular niche.

In *Aspergillus fumigatus* infection, macrophages, particularly alveolar macrophages, are essential for early containment through uptake of inhaled conidia [32,38]. However, when intracellular killing is incomplete, viable conidia can persist and germinate under conditions of impaired immunity, transforming a controlled encounter into a trajectory toward invasive disease [32,38]. Collectively, macrophages act as both antifungal effectors and intracellular reservoirs. The balance between fungicidal activity, inflammatory regulation, and pathogen-driven subversion determines whether infection is controlled or progresses, positioning macrophages centrally within the damage–response continuum.

## 3. Mesenchymal Stem Cells: Core Immunomodulatory and Reparative Functions

### 3.1. Canonical Properties of MSCs

Mesenchymal stem/stromal cells (MSCs) are multipotent stromal cells with self-renewal and differentiation capacity, as defined by International Society for Cellular Therapy criteria [54]. However, across most therapeutic contexts, their dominance is attributed to context-responsive paracrine immunomodulation rather than durable engraftment or direct tissue replacement [17,54,55]. Accordingly, MSCs act primarily through their secretome—a complex repertoire of cytokines, chemokines, growth factors, and extracellular vesicles (including exosomes)—that modulates immune activation, leukocyte trafficking, angiogenesis, and extracellular matrix remodeling, although its composition is not uniform and may vary across condition [56,57]. The importance of vesicle-mediated signaling is supported by experimental studies showing that MSC-derived exosomes recapitulate key paracrine functions in vivo [18]. While MSCs are best known for roles in regeneration and immune regulation, recent work has increasingly examined how these same immunoregulatory mechanisms operate within infectious microenvironments, including fungal disease [19].

### 3.2. MSC Interactions with Innate and Adaptive Immunity

MSCs are known to modulate immune responses across both innate and adaptive compartments [58,59,60,61,62]. Within the innate compartment, MSCs can regulate macrophage activation and polarization, dampening pro-inflammatory programs while promoting regulatory/pro-resolving states; experimental studies combining in vivo and in vitro models have shown that this shift is associated with COX-2/PGE2-dependent changes in macrophage phenotype and increased IL-10 production [58,59]. MSCs can also influence neutrophils by altering recruitment and survival kinetics and have been shown to inhibit NK cell proliferation, cytotoxicity, and IFN-γ production [63,64,65]. Importantly, MSCs modulate dendritic cells (DCs), which bridge innate and adaptive immunity by inhibiting DC differentiation/maturation, reducing antigen presentation and co-stimulation (e.g., MHC II, CD80/CD86), and thereby limiting T-cell activation [60,65].

Within the adaptive compartment, MSCs have been shown to suppress T-cell proliferation and effector differentiation, often reducing Th1/Th17 responses while promoting expansion/induction of FoxP3^+^ regulatory T cells (Tregs) [61,65]. MSCs also inhibit B-cell proliferation, plasma–cell differentiation, and antibody production under inflammatory conditions [62]. Overall, MSCs dampen T-cell-driven inflammation and promote regulatory responses, but the extent of this shift depends on the inflammatory signals present in the tissue at the time MSCs interact with immune cells. While these immunomodulatory effects may limit excessive inflammation, they may also impair antifungal host defense depending on the infectious context.

## 4. MSC–Macrophage Crosstalk in Fungal Contexts

### 4.1. Modulation of Macrophage Inflammatory Activation

Direct mechanistic evidence for MSC–macrophage crosstalk in fungal settings remains limited, with only a few primary studies interrogating macrophage responses to fungal pathogens or fungal-derived ligands in the presence of MSCs. In an in vitro murine co-culture model, Cho et al. (2016) [66] reported that MSCs alter macrophage responses to *Aspergillus fumigatus* conidia, shifting the TNF-α/IL-10 axis and suggesting involvement of NF-κB-associated pathways in fungal control. Supportive mechanistic evidence also comes from indirect models. In a complementary ligand-based model, Choi et al. (2011) [67] described a TSG-6–CD44 pathway through which MSCs can attenuate zymosan/TLR2-driven NF-κB activation and restrain macrophage inflammatory amplification in vivo, providing a defined mechanistic framework for tuning inflammatory set-points downstream of fungal cell wall sensing. Importantly, these few available studies remain heterogeneous in pathogen, model, and experimental design, making broader mechanistic conclusions difficult.

### 4.2. Effects on Macrophage Phagocytosis and Fungal Uptake

Beyond inflammatory modulation, an important unresolved question is whether MSCs modulate macrophage-mediated fungal uptake, intracellular processing, and fungal clearance. This distinction is especially important in fungal pathogenesis, where phagocytosis does not necessarily culminate in fungal elimination but may instead result in persistence within host cells.

In an in vitro human macrophage model, Holopainen et al. (2020) reported that MSC secretome exposure increased CD206 expression and was accompanied by enhanced *Candida albicans* phagocytosis [68].

However, this study primarily supports altered uptake and immunophenotype; it does not establish improved intracellular killing. Indeed, increased phagocytosis coincided with induction of immunoregulatory markers such as PD-L1 and MerTK, raising the possibility that enhanced internalization and restrained inflammatory activation may coexist without necessarily improving fungicidal efficacy.

In mold infection, macrophage uptake is likewise critical at early stages, particularly in the lung, where alveolar macrophages internalize inhaled *Aspergillus conidia*. Here, outcomes hinge on whether intracellular killing prevents persistence and germination. Consistently, reduced fungal growth in *Aspergillus conidia* co-culture systems [66] suggests that MSCs may influence macrophage-mediated fungal uptake and early antifungal responses, although direct evidence regarding phagosomal maturation, fungicidal mechanisms, and long-term intracellular fungal fate remains limited.

Overall, current data support the possibility that MSC-derived programs alter fungal uptake while constraining inflammatory amplification. However, the evidence remains insufficient to conclude that MSCs consistently improve intracellular fungal killing. A central unresolved question is whether MSC-induced macrophage function promotes fungicidal maturation or, under some conditions, favors intracellular persistence.

## 5. Direct Interactions Between MSCs and Fungal Pathogens

### 5.1. Fungal Sensing and Internalization by MSCs

Beyond indirect immune modulation, MSCs have been shown to recognize fungal cells and PAMPs via PRRs (TLR2, TLR4, and Dectin-1), and establish physical contact with pathogens [69,70,71]. MSC–fungus interactions are often accompanied by phagocytosis-like uptake; however, this capacity is limited compared to professional phagocytes (e.g., macrophages and neutrophils), suggesting that uptake alone is unlikely to represent a dominant fungicidal mechanism [19,71]. This is consistent with the more limited antimicrobial capacity of MSCs compared to professional phagocytes.

Although fungal recognition is a common early event, downstream consequences vary widely across models—from neutral effects to antifungal activity mediated by soluble factors—depending on fungal species, MSC source/state, and inflammatory context [66,67,68]. For example, in an in vitro model of *Paracoccidioides brasiliensis* and *Histoplasma capsulatum*, the consequences are characterized by fungal internalization without effective clearance, with *H. capsulatum* additionally compromising MSC viability and function [68,69].

Evidence from experimental bacterial infection models suggests that MSCs contribute to antimicrobial defense through the secretion of antimicrobial peptides (AMPs), including LL-37/cathelicidin and β-defensins, as well as other soluble mediators [72,73], supporting the possibility of similar mechanisms in fungal settings (see Section 5.3).

### 5.2. Signaling Responses to Fungal Components

Several studies suggest that MSCs respond to isolated fungal components even in the absence of live organisms. In an in vivo allergic airway model, exposure to *Aspergillus* hyphal extract showed that MSC efficacy may depend on inflammatory context and stage, with beneficial effects in acute inflammation but limited impact in chronic setting [74].

At the signaling level, Tidu et al. (2021) [75] showed in an in vitro study that zymosan (a β-glucan-rich fungal cell wall preparation) is phagocytosed by MSCs and activates PRR-driven pathways in MSCs, including Syk phosphorylation, increased cytosolic Ca^2+^, and downstream transcriptional responses coupled to inflammatory mediator production (e.g., IL-6, IL-8). These findings suggest that MSCs may function as fungal PAMP-responsive stromal sentinels, translating fungal signals into inflammatory and tissue-remodeling programs [72,75]. However, whether these signaling responses translate into effective anti-fungal activity remains unclear. Overall, MSC effects appear strongly context- and stage-dependent: they may be more beneficial during early/acute inflammation and less effective (or qualitatively different) once chronic inflammatory and fibrotic pathways are established.

### 5.3. Pathogen-Specific Outcomes

In *Candida albicans* models, Yang et al. (2013) [76] reported an IL-17-producing MSC subset capable of inhibiting fungal growth in vitro and reducing fungal burden in vivo, independent of classical phagocytosis, although whether this reflects true antifungal activity or growth restriction without clearance remains unsolved. Notably, this subset appeared to lose canonical immunosuppressive properties while acquiring antifungal activity. However, direct MSC-mediated phagocytosis was not demonstrated in that study.

More recently, Bicer et al. (2025) [77] reported that conditioned medium (CM) from 3D-cultured palatal MSCs can inhibit *Candida* growth through secreted antimicrobial peptides, highlighting a central role for soluble mediators in MSC antifungal activity. Among these, the cathelicidin peptide LL-37 emerged as a major contributor to the observed effect [73,77]. Notably, the enhanced antifungal activity under 3D culture conditions suggests that spatial organization and microenvironmental cues critically shape how MSCs respond functionally to fungal pathogens.

In *Aspergillus fumigatus* models, MSCs internalize conidia and reduce CFU without impairing cytokine secretion, oxidative burst, or T-cell activation, suggesting no overt impairment of selected antifungal effector functions in that experimental system [71].

Collectively, the limited available studies of *Candida albicans* and *Aspergillus fumigatus* models do not consistently show overt worsening of infection following MSC exposure. However, these findings should be interpreted cautiously. The number of studies remains small, the experimental systems are heterogeneous, and model-specific conditions—including fungal burden, timing of MSC administration, host immune context, and readouts used to define benefit or harm—may substantially influence the observed outcome. Accordingly, the absence of reported detrimental effects in these models should not be interpreted as evidence of safety. Rather, current data suggest that MSC effects in *Candida* and *Aspergillus* settings remain incompletely defined and may be strongly model-dependent. Notably, studies in dimorphic fungal infections reveal divergent outcomes. In a chronic murine model of *Paracoccidioides brasiliensis* infection, MSC administration exacerbated pulmonary fibrosis and increased fungal burden, potentially through skewing immune responses toward non-protective, pro-repair states [78,79]. Arango et al. (2017) [79] reported increased fibrocyte accumulation, higher collagen deposition, and upregulation of pro-fibrotic genes, including *Col3α1*, *TGF-β3*, *MMP-8*, and *MMP-15*. In a follow-up study, Arango et al. (2018) [78] showed that MSC treatment altered the immune landscape toward a non-protective profile, characterized by increased M2 and decreased M1 macrophages, alongside increased inflammatory cell infiltration and fungal burden.

Similarly, an in vitro study showed that MSCs internalized *Histoplasma capsulatum* but failed to exert fungicidal activity, underwent apoptosis, and exhibited functional impairment, suggesting the potential formation of permissive niches for persistence. The infection was associated with increased IL-6 expression and decreased expression of *TGF-β*, *IL-17*, *IL-1β*, *TNF-β*, *iNOS* and *Arg-1* [70]. Overall, MSC effects in fungal infection appear strongly pathogen- and context-dependent. Although studies in *Candida albicans* and *Aspergillus fumigatus* models have generally not reported overt detrimental effects, the available evidence remains limited and should be interpreted cautiously. In chronic dimorphic fungal infections, by contrast, MSC-driven immune regulation and repair programs may favor non-protective reparative responses over pathogen clearance, thereby promoting persistence and progressive fibrosis. Thus, similar to macrophages, MSCs may, under specific inflammatory conditions, shift from protective regulators to permissive cellular environments that support fungal persistence. This context dependence extends beyond fungal control itself to tissue repair after infection. For example, Zhou et al. (2019) [80] investigated whether uMSCs, administered after antifungal treatment with natamycin, could modulate disease outcome in a murine model of *Fusarium oxysporum* keratitis, and showed reduced corneal fibrosis and opacity, associated with inhibition of TGF-β1/Smad2 signaling, without evidence of direct antifungal activity.

## 6. The MSC Secretome as a Potential Cell-Free Adjunct in Fungal Disease

### 6.1. From Cell Therapy to Programmable Paracrine Intervention

Accumulating evidence across Section 4 and Section 5 suggests that many MSC effects in fungal settings are mediated by secreted factors that reshape immune set-points and modulate antifungal host responses, rather than primarily through durable engraftment or direct cellular killing. These observations support the view that MSCs act, at least in part, through a paracrine secretome that may influence the balance between fungal clearance and host-mediated tissue injury. However, in invasive fungal infections, the translational relevance of this concept remains incompletely defined, and the available evidence is still limited. In IFIs, this distinction is particularly relevant, as patients are often profoundly immunocompromised and additional systemic immune suppression carries risk. A cell-free approach has been proposed as a strategy to enable controlled dosing, defined composition, and reduced persistence-related safety concerns, while potentially retaining the immunoregulatory and antimicrobial properties of MSC-derived mediators [19]. However, these approaches remain largely speculative in invasive fungal disease, and their translational relevance is still insufficiently defined.

### 6.2. Mapping Secretome Effects onto the Damage–Response Framework

Fungal disease progression reflects a dynamic imbalance between pathogen burden and host-mediated injury. The MSC secretome may influence both axes: pathogen burden and host-mediated tissue injury.

#### 6.2.1. Modulation of Inflammatory Set-Points

MSC-derived mediators have been reported to modulate macrophage inflammatory programs downstream of PRR engagement. This includes a shift in the TNF-α/IL-10 balance and attenuation of NF-κB-linked amplification [66,67]. Importantly, this modulation may dampen excessive inflammation while preserving the host’s ability to respond to fungal infection. However, evidence that such inflammatory recalibration translates into improved fungal clearance, survival benefit, or reduced tissue fungal burden in vivo remains limited.

This “set-point tuning” may be particularly relevant in mold infections where inflammasome activation and hyphae-driven signaling contribute substantially to tissue injury (Section 2). By potentially limiting cytokine amplification while preserving phagocytic activity, MSC-derived factors may reduce collateral damage. Whether they also preserve fungal control remains unclear. Overall, these effects are still insufficiently tested across fungal settings.

#### 6.2.2. Effects on Fungal Uptake and Intracellular Fate

Enhanced macrophage uptake of *Candida albicans* in the presence of MSC-conditioned medium [67] suggests that secreted factors may influence early recognition and internalization, potentially via receptors such as CD206. However, increased uptake is not equivalent to improved fungal clearance. For several IFI pathogens, intracellular persistence within macrophages is itself a central pathogenic mechanism [66]. The key mechanistic question is therefore not whether MSC-derived factors enhance uptake, but whether they promote fungicidal maturation, including phagolysosomal fusion, LC3-associated processing, ROS-dependent killing, and durable suppression of intracellular fungal viability. At present, these endpoints remain largely unmeasured in most MSC–fungus systems. Consequently, it remains unclear whether MSC- or secretome-induced modulation of macrophage function improves intracellular fungal control or, under some conditions, stabilizes permissive intracellular niches. This distinction is particularly important when interpreting the translational promise of MSC-derived secretome products. A strategy that enhances uptake while simultaneously dampening inflammatory or fungicidal pathways could have very different consequences depending on fungal species, immune context, and the intrinsic ability of macrophages to complete intracellular killing. For this reason, future studies should move beyond uptake assays alone and incorporate mechanistic readouts that more directly assess intracellular fungal fate.

#### 6.2.3. Tissue Repair Versus Permissive Remodeling

In murine models of dimorphic fungal infections, MSC administration has been associated with pro-fibrotic remodeling and increased fungal burden [78,79]. These findings raise the possibility that paracrine repair programs relevant to MSC activity may become maladaptive when pathogen persistence requires sustained fungicidal pressure. This duality mirrors macrophage biology itself: pro-resolving states may limit inflammation yet create permissive conditions for chronic infection. Therefore, the effects of MSC-associated immunomodulatory programs likely depend on timing, fungal species, and host immune status. Acute inflammatory settings may benefit from controlled immunomodulation, whereas chronic granulomatous or fibrotic disease may not.

### 6.3. Antifungal Effector Components Within the Secretome

Although MSCs primarily act through immunomodulation, a limited number of secreted components have shown antifungal activity in experimental systems. Conditioned medium from 3D-cultured MSCs inhibits *Candida albicans* growth, with LL-37 identified as a key mediator [77]. LL-37 can disrupt fungal membrane and may modulate immune recruitment. Similarly, an IL-17-producing MSC subset exhibited antifungal activity independent of classical phagocytosis [76].

Other antimicrobial molecules within the MSC secretome—including β-defensin 2, hepcidin, lipocalin, lysozyme, and Histatin-5—may contribute to antifungal defense [19]. However, direct evidence for their activity in invasive fungal models remains limited, highlighting the need to distinguish demonstrated effects from predicted activity. Importantly, these effects are supported by limited experimental systems, whereas many proposed antifungal roles of the MSC secretome remain extrapolated and require further validation.

### 6.4. Extracellular Vesicles as a Candidate Therapeutic Fraction

Extracellular vesicles (EVs), including exosomes, represent a concentrated and potentially standardizable component of the MSC secretome, although their heterogeneity remains a major challenge. Owing to their small size, exosomes can penetrate tissue and traverse biological barriers, (e.g., the blood–brain barrier), which may be relevant in IFI with limited drug or immune access [19,81]. Exosomes from TLR4-primed MSCs have been reported to skew immune responses toward a pro-inflammatory profile associated with enhanced antifungal activity and improved clearance [81]. However, these findings remain limited, and their relevance across fungal pathogens and clinical IFI settings is unclear. More broadly, EV-specific antifungal efficacy data remain sparse, with key unresolved questions regarding biodistribution in systemic IFI, persistence and biological activity within infected tissues, and effects on intracellular fungal survival.

## 7. Graft-Versus-Host Disease, Fungal Infection, and MSC Therapy

### 7.1. GvHD as a High-Risk Setting for Invasive Fungal Infection

Graft-versus-host disease (GvHD) following allogeneic hematopoietic cell transplantation is among the high-risk contexts for invasive fungal infections (IFIs). Susceptibility reflects multiple factors, including mucosal barrier disruption, prolonged corticosteroid exposure, lymphopenia, and cumulative immunosuppression from second-line therapies. Steroid-refractory acute GvHD (SR-aGvHD) is particularly associated with increased invasive mold infections and infection-related mortality. Thus, GvHD represents a clinically relevant “stress test” for immunomodulatory interventions, in which benefits in immune regulation must be evaluated alongside potential effects on antifungal host defense.

### 7.2. MSC Therapy for Steroid-Refractory GvHD

MSCs are widely used as second-line therapy for steroid-refractory GvHD, although efficacy remains heterogeneous [73,82]. A phase 3 trial of remestemcel-L did not show superiority to placebo [82], whereas subsequent studies reported reduced biomarkers of tissue injury and immune activation following MSC treatment [83,84,85]. Combination approaches (e.g., with basiliximab or calcineurin inhibition) and meta-analyses suggest improved responses [83] and failure-free survival in steroid-refractory disease [84,85].

Clinical experience, including efficiency patterns and infection outcomes, has been synthesized by Zhao et al. (2019) [86]. Emerging platforms, including iPSC-derived MSCs [86], further expand the therapeutic landscape [87]. Beyond whole-cell therapy, MSC-derived extracellular vesicles are being explored as alternative modalities with potential advantages in product standardization and safety control [88,89].

Given the profound baseline susceptibility to opportunistic infections in SR-GvHD, the key unresolved question is not only whether MSCs control inflammation, but also how MSC therapy influences IFI risk.

### 7.3. Invasive Fungal Infection Risk During MSC Therapy

Early concerns suggested that MSC-mediated immunomodulation might increase susceptibility to opportunistic infections [90]. However, interpretation of the clinical literature remains difficult because MSCs have often been administered in late-line or salvage settings to critically ill patients with severe steroid-refractory GvHD, profound immune dysfunction, and substantial prior exposure to corticosteroids and other immunosuppressive agents, often in the setting of variable antifungal prophylaxis. In such cohorts, baseline infectious risk is already high, making causal attribution to MSC therapy particularly challenging [91].

More recent studies suggest that treatment, timing and context may influence observed outcomes. Earlier or pre-emptive MSC strategies have been associated with improved GvHD control and reduced corticosteroid exposure, without a clear signal for increased fungal infection rates or infection-related mortality in the available studies [92,93]. Sequential infusion protocols similarly improved survival without increasing non-relapse mortality [93], and meta-analyses suggest that MSC administration does not increase overall infection risk [19,92].

However, fungal-specific risk remains unresolved. Some studies report increased IFI incidence in severe steroid-refractory disease [73,94], whereas other studies report no significant increase in infection-related mortality with MSC treatment [95,96]. In some cohorts, MSC recipients receiving prophylaxis experienced fewer severe infections, although this was not stratified by pathogen [83]. Other studies found reduced GvHD without a parallel reduction in infectious complications [97], while increased IFI risk has been observed in severe cases [94].

Meta-analytic data do not clearly support an association between MSC therapy and increased overall infection risk [98]. However, these findings should be interpreted cautiously because of substantial confounding and heterogeneity across studies. Some analyses have also suggested reduced infection-attributable mortality, although fungal-specific adjudication is frequently lacking [86,98].

Experimental data provide only limited indirect reassurance. In one in vitro study, Schmidt et al. showed that human MSCs did not overtly impair selected anti-*Aspergillus fumigatus* host defense readouts, including anti-*Aspergillus* CD4+ T-cell effector function and neutrophil oxidative burst activity, while higher MSC-to-conidia ratios were associated with reduced fungal colony formation [71]. However, these findings derive from a controlled experimental model and should not be interpreted as evidence of clinical safety in profoundly immunocompromised patients.

Taken together, current clinical data do not allow for firm conclusions regarding the specific effect of MSC therapy on invasive fungal infection risk, given the substantial overlap with disease severity, concurrent immunosuppressive treatments, and inconsistent pathogen-specific reporting.

### 7.4. Confounding Factors and Interpretation Challenges

Assessment of infection risk during MSC therapy is complicated by multiple confounders. MSCs are frequently administered to patients with severe GvHD, prolonged and/or prior immunosuppression, and advanced disease, all of which independently increase infection susceptibility [73,82]. Interpretation is further complicated by the timing of MSC administration, which often differs between early/pre-emptive and late salvage settings, and by variation in antifungal prophylaxis across studies [73,84,91]. In addition, improved survival may increase observed infection incidence, as patients live longer and remain exposed to infectious risks over extended periods [98].

Many studies also lack pathogen-specific stratification, limiting conclusions for IFIs [85,87,91,95,97]. These limitations substantially restrict the ability to draw definitive conclusions and highlight the need for fungal-specific clinical endpoints, standardized prophylaxis reporting, and mechanistic biomarkers in future MSC trials.

To integrate the evidence discussed across in vitro, in vivo, and clinical settings, including GvHD-associated human studies, Table 1 summarizes the principal reported MSC effects across fungal pathogens and related models.

## 8. Conceptual Integration: When Do MSCs Help or Harm?

Across experimental and clinical contexts, MSCs primarily function as regulators of immune balance rather than direct antifungal effectors. Their benefits are most evident in inflammation-dominated settings, where limiting host-mediated tissue injury improves outcomes. Conversely, in chronic or poorly controlled infections—particularly those requiring sustained cellular immunity for clearance (e.g., intracellular or dimorphic fungi)—MSC-driven immune regulation and repair programs may inadvertently favor pathogen persistence [8,10,89].

Overall, the available evidence supports a model in which MSC-derived soluble factors may modulate macrophage function while constraining excessive inflammation, although their effects on fungal clearance remain incompletely defined. This interpretation is broadly consistent with the limited available *Candida albicans* and *Aspergillus fumigatus* models, in which overt detrimental effects have not been consistently observed, but the evidence base remains sparse and should not be interpreted as evidence of safety. By contrast, chronic dimorphic fungal infections more clearly illustrate that MSC-driven immune regulation and reparative programs may favor non-protective responses over effective pathogen clearance, thereby promoting persistence and fibrosis [70,79,99].

**Figure 1:**
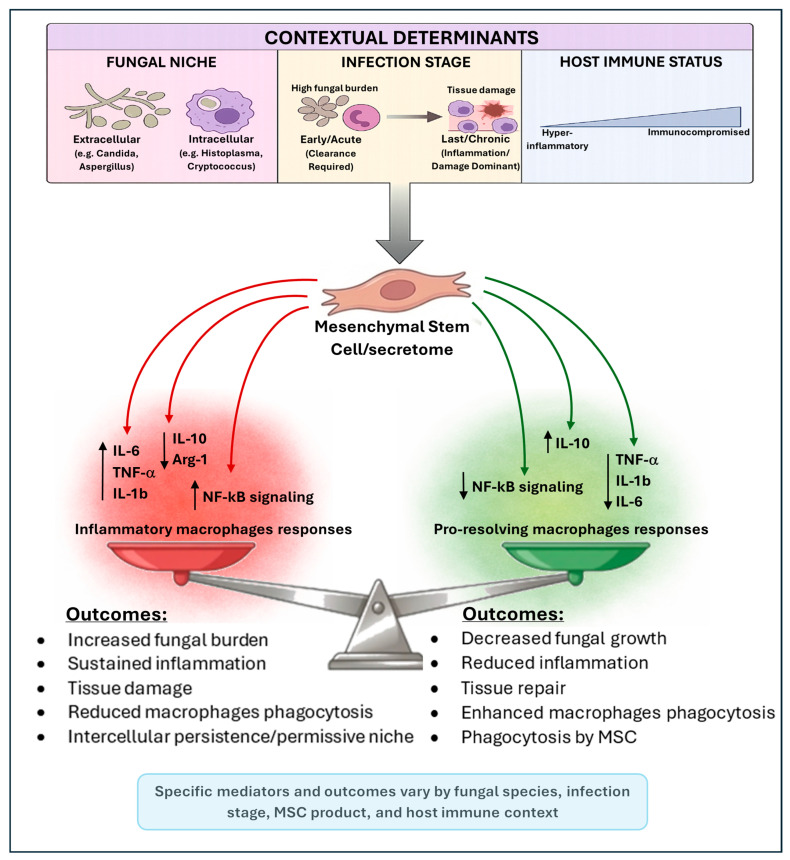
Context-dependent effects of mesenchymal stem cells (MSCs) in fungal infections. Mesenchymal stem cells (MSCs) and their secretome exert dual, context-dependent immunomodulatory effects during fungal infections. Outcomes are shaped by key determinants, including the fungal niche (extracellular pathogens versus intracellular pathogens), infection stage (early/acute clearance versus late/chronic inflammation), and host immune status. MSCs can promote pro-resolving macrophage responses, associated with increased Interleukin-10 (IL-10); reduced Tumor Necrosis Factor alpha (TNF-α), Interleukin-1 beta (IL-1β), and Interleukin-6 (IL-6); and inhibition of Nuclear Factor kappa B (NF-κB) signaling, leading to improved phagocytosis, reduced fungal growth, and tissue repair. Conversely, MSCs may sustain inflammatory responses, characterized by increased IL-6, TNF-α, and IL-1β; activation of NF-κB signaling; and reduced IL-10 and Arginase-1 (Arg-1), resulting in impaired fungal clearance, persistence, and tissue damage. Mediators and outcomes shown are selected examples and not exhaustive, highlighting the context-dependent plasticity of MSC effects in fungal infections.

Although MSCs can internalize fungi, their phagocytic capacity is far lower than that of professional phagocytes, and whether they effectively kill internalized pathogens remains unclear [19]. Overall, MSC actions appear to be largely mediated through secreted factors and immunomodulation, reinforcing their context dependence [57,77,99]. This context-dependent framework, summarized in Figure 1, may support stratifying future MSC trials by infection phenotype, immune status, and fungal pathogen class, while incorporating fungal-specific endpoints and mechanistic biomarkers. 

## 9. Conclusions and Future Directions

MSCs represent a context-dependent host-directed strategy in fungal disease. Their dominant contribution appears to be immunomodulation—particularly shaping macrophage function and inflammatory resolution—rather than direct pathogen killing. Accordingly, optimizing MSC source, timing, and delivery alongside secretome-based approaches may be critical for safe translation.

Key translational gaps include standardization (donor variability, tissue source, culture format, and priming), fit-for-purpose potency assays (fungal burden/CFU, intracellular killing, macrophage calibration, and tissue injury readouts beyond cytokines), timing (acute versus chronic/fibrotic IFI) and safety in severely immunocompromised hosts such as GvHD, where subtle impairment of antifungal defense may be difficult to detect.

Overall, the MSC secretome is best viewed as a context-dependent immunoregulatory platform intersecting fungal pathogenesis at multiple checkpoints. Defining conditions under which MSC-driven immunoregulation promotes clearance rather than persistence will be essential to advancing safe, pathogen-informed antifungal adjuncts.

## Figures and Tables

**Table 1:** Summary of studies evaluating MSC effects in fungal infection and related fungal-response models.

Number	Paper	Model	Fungi	MSC	MSC Effect
1	Cho et al. (2016) [66]	In vitro (murine macrophages—J774A.1 cells)	*Aspergillus fumigatus*	Murine bone marrow-derived MSCs	Immunomodulation: MSC decreased TNF-a and increased IL-10 in macrophages and reduced NF-kB signaling Inflammatory response: direct effect—fungal growth decreased
2	Choi et al. (2011) [67]	In vivo (murine) and in vitro (murine macrophage-like cell line— RAW 264.7, human mesothelial cells—MeT- 5A, ATCC and human embryonic kidney 293 cells)	Zymosan	Human bone marrow-derived MSCs	Immunomodulation: MSC via TSG-6 decreased TNF-a, IL-1b, IL-6, NF-kB in macrophages Tissue effect: decreased in neutrophil recruitment Direct effect: reduce inflammation
3	Holopainen et al. (2020) [68]	In vitro (human peripheral blood mononuclear cells)	*Candida albicans*	Human bone marrow-derived MSC secretome	Immunomodulation: increased in IL-10, macrophage polarization (increased in CD206 and PD-L1) Direct effect (via macrophages): increased in phagocytosis Inflammatory response: improved macrophage phagocytosis
4	Rodriguez-Echeverri et al. (2021) [69]	In vitro (murine bone marrow- derived MSCs)	*Paracoccidioides* *brasiliensis*	Murine bone marrow-derived MSCs	Immunomodulation: increased in IL-6, TNF-a, IL-17, TGF-b Direct effect (MSC): internalization without killing Harmful: impaired fungal control
5	Rodríguez-Echeverri et al. (2023) [70]	In vitro (murine bone marrow- derived MSCs)	*Histoplasma* *capsulatum*	Murine bone marrow-derived MSCs	Immunomodulation: increased in IL-6, decreased in TGF-b, IL-17, IL-1b, TNF-a, iNOS, Arg-1 Direct effect (MSC): internalization without killing Harmful: apoptosis, decrease in proliferation, impaired differentiation
6	Schmidt et al. (2017) [71]	In vitro (human bone marrow- derived MSCs)	*Aspergillus fumigatus*	Human bone marrow-derived MSCs	Immunomodulation: no change in cytokine secretion (TNF-a, IL-6, IL-1b), no effect on oxidative burst/T cells Direct effect (MSC): phagocytosis of conidia—decrease in CFU, phagocytosis inhibition abolishes MSC effect
7	Lathrop et al. (2014) [74]	In vivo (murine)	*Aspergillus fumigatus*	Murine bone marrow-derived MSCs	Immunomodulation: decreased in IL-17 and Th17 Tissue effect: decreased in airway inflammation Direct effect: beneficial effect in acute inflammation, limited impact in chronic setting
8	Tidu et al. (2021) [75]	In vitro (human bone marrow- derived MSCs)	Zymosan	Human bone marrow-derived MSCs	Immunomodulation: increased in IL-6, IL-8, IL-1b, IL-2, IL12P40, IDO (NF-kB-dependent) Direct effect: phagocytosis of zymosan with phagosome acidification Tissue effect: ECM remodeling (NFAT-dependent)
9	Yang et al. (2013) [76]	In vivo (murine) and in vitro (Tregs and Th17 cells)	*Candida albicans*	Murine + human bone marrow-derived MSCs (IL17^+^ subset)	Immunomodulation: failed to upregulate Treg and failed to downregulate Th17 cells, decreased IL-10 and TGF-b Tissue effect: improved fungal clearance and tissue recovery Direct effect: IL-17-mediated antifungal activity Harmful: impaired immunosuppression function via NF-kB-mediated downregulation of TGF-b
10	Bicer et al. (2025) [77]	In vitro (human palatal adipose tissue- derived MSCs)	*Candida albicans*	Human palatal adipose tissue-derived MSCs (PAT-MSCs) and MSCs secretome	Direct effect: MSC inhibited fungal growth (CFU reduction), MSC secretome (CM) inhibited fungal growth with comparable efficiency, combination of MSCs and CM showed synergistic antifungal activity, IL-37-mediated antifungal activity
11	Arango et al. (2017) [79]	In vivo (murine)	*Paracoccidioides* *brasiliensis*	Murine bone marrow-derived MSCs	Immunomodulation: increased pro-fibrotic genes (TGF-b3, collagen, MMPs) Tissue effect: increased collagen deposition, fibrotic accumulation and pulmonary fibrosis Harmful: MSC failed to exert anti-fibrotic activity
12	Arango et al. (2018) [78]	In vivo (murine)	*Paracoccidioides* *brasiliensis*	Murine bone marrow-derived MSCs	Immunomodulation: increased IL-6, IL-9, GM-CSF, CXCL1, CXCL9, CCL5, increased M2 macrophages and decreased M1 macrophages and Treg Tissue effect: increased pulmonary inflammation and granulomatous response Harmful: fungal burden increased and no pathogen clearance
13	Zhou et al. (2019) [80]	In vivo (murine)	*Fusarium oxysporum*	Humen umbilical cord-derived MSCs	Immunomodulation: increased inflammatory cell infiltration and downregulation of TGF-b1/Smad signaling Tissue effect: reduced corneal fibrosis, opacity and scar formation Direct effect: no direct antifungal activity (effect observed in combination with antifungal treatment)
14	Kebriaei et al. (2020) [82]	GvHD clinical setting	Invasive fungal infections (*Candida*, *Aspergillus*)	Human bone marrow-derived MSCs	Immunomodulation: systemic immunosuppression associated with MSC therapy in GvHD Tissue effect: improved clinical response in GvHD patients Harmful: fungal infections reported as adverse events, with no clear increase compared to control (no causal relationship established)
15	Zhao et al. (2022) [85]	GvHD clinical setting	Not specific (fungal infections reported as part of overall infections)	Human bone marrow-derived MSCs	Immunomodulation: increased overall response rate and sustained clinical response in SR-aGvHD Tissue effect: reduced treatment-related toxicity Harmful: fungal infections reported as adverse events
16	Bloor et al. (2020) [87]	GvHD clinical setting	Not specific	iPSC-derived MSCs	Direct effect: increased overall response and survival, improved GvHD severity
17	Le Blanc et al. (2008) [91]	GvHD clinical setting	Not specific	Humen umbilical cord-derived MSCs	Immunomodulation: significantly reduced incidence and severity of aGvHD, improved GvHD-free and relapse-free survival (GRFS)
18	Ning et al. (2008) [97]	GvHD clinical setting	Not specific	Human bone marrow-derived MSCs	Immunomodulation: reduced incidence and severity of GvHD Harmful: increased relapse rate
19	Weng et al. (2010) [95]	GvHD clinical setting	Not specific	Human bone marrow-derived MSCs	Immunomodulation: increased in CD4+ T cells and B-cell subset, decreased in CD8+ T cells Direct effect: increased overall response and survival, improved GvHD severity

The table summarizes in vitro, in vivo, and clinical studies, including GvHD-associated human data, and outlines the reported immunomodulatory, direct antifungal, tissue-level, and potentially deleterious effects of MSCs or MSC-derived products across different fungal pathogens and model systems.

## Data Availability

No new data were created or analyzed in this study. All information presented in this review is derived from previously published studies cited in the article.
